# Comparative Genome Analysis of the Closely Related *Synechocystis* Strains PCC 6714 and PCC 6803

**DOI:** 10.1093/dnares/dst055

**Published:** 2014-01-09

**Authors:** Matthias Kopf, Stephan Klähn, Nadin Pade, Christian Weingärtner, Martin Hagemann, Björn Voß, Wolfgang R. Hess

**Affiliations:** 1 Genetics and Experimental Bioinformatics, Faculty of Biology, University of Freiburg, Schänzlestr. 1, D-79104 Freiburg, Germany; 2 Plant Physiology, Institute for Life Sciences, University of Rostock, Einsteinstr. 3, D-18059 Rostock, Germany

**Keywords:** comparative genomics, cyanophages, genome sequence, prophage, salt acclimation

## Abstract

*Synechocystis* sp. PCC 6803 is the most popular cyanobacterial model for prokaryotic photosynthesis and for metabolic engineering to produce biofuels. Genomic and transcriptomic comparisons between closely related bacteria are powerful approaches to infer insights into their metabolic potentials and regulatory networks. To enable a comparative approach, we generated the draft genome sequence of *Synechocystis* sp. PCC 6714, a closely related strain of 6803 (16S rDNA identity 99.4%) that also is amenable to genetic manipulation. Both strains share 2838 protein-coding genes, leaving 845 unique genes in *Synechocystis* sp. PCC 6803 and 895 genes in *Synechocystis* sp. PCC 6714. The genetic differences include a prophage in the genome of strain 6714, a different composition of the pool of transposable elements, and a ∼40 kb genomic island encoding several glycosyltransferases and transport proteins. We verified several physiological differences that were predicted on the basis of the respective genome sequence. Strain 6714 exhibited a lower tolerance to Zn^2+^ ions, associated with the lack of a corresponding export system and a lowered potential of salt acclimation due to the absence of a transport system for the re-uptake of the compatible solute glucosylglycerol. These new data will support the detailed comparative analyses of this important cyanobacterial group than has been possible thus far. Genome information for *Synechocystis* sp. PCC 6714 has been deposited in Genbank (accession no AMZV01000000).

## 1. Introduction

Genomic and transcriptomic comparisons between closely related bacteria are powerful approaches to infer insight into the metabolic potentials and regulatory networks. Among cyanobacteria, this has been illustrated by detailed comparative analyses of the marine picoplanktonic cyanobacteria *Prochlorococcus* and *Synechococcus.*^1–3^ However, due to the lack of data from closely related strains, no comprehensive comparison has focused on *Synechocystis* sp. PCC 6803 (from here on *Synechocystis* 6803), the otherwise most popular cyanobacterial system to work with. *Synechocystis* 6803 was the first phototrophic and the third organism overall for which a complete genome sequence was determined.^4^ The genome of *Synechocystis* 6803 was manually curated by the research community at CyanoBase (http://genome.microbedb.jp/cyanobase/Synechocystis).^5^ Over the years, several substrains of 6803 evolved in different laboratories showing distinct physiological features (e.g. glucose tolerance), from which also several have recently been re-sequenced.^6–9^

The coverage with analysed genome sequences for the cyanobacterial phylum has been greatly improved recently. Based on a diversity-driven selection of species for genome sequencing, 54 additional strains were analysed,^10^ raising the number of publicly available cyanobacterial genome sequences to 126. With strain PCC 7509 also, one *Synechocystis* strain was sequenced. However, it is only very remotely related (90% 16S rRNA identity) to *Synechocystis* 6803 and belongs even to another clade (B1) than *Synechocystis* 6803 (B2) in the cyanobacterial tree.^10^ Therefore, despite its naming as *Synechocystis*, the strain PCC 7509 is quite distant from *Synechocystis* 6803. In the current cyanobacterial tree, *Synechocystis* 6803 is sharing a clade with unicellular N_2_-fixing oceanic strains such as *Cyanothece* spp.^10^ It has been reported that a 97–100% 16S rRNA identity is necessary for a productive genome comparison among strains.^1–3^

Thus, *Synechocystis* 6803 lacked a closely related organism with a known genome sequence that appeared suitable for comparative analysis. To fill this gap, we selected *Synechocystis* sp. PCC 6714 (from here: *Synechocystis* 6714) as candidate. *Synechocystis* 6803 as well as strain 6714 are unicellular cyanobacteria that were isolated from the same freshwater pond in Oakland, California, by R. Kunisawa. These strains were initially part of the ‘Berkeley Culture Collection’,^11^ which were later transferred into the ‘Pasteur Culture Collection’ of cyanobacteria.^12^ The decision to choose *Synechocystis* 6714 was further supported by the high 16S rRNA identity (99.4%) among the two strains, thus well suited for comparative analyses. Their close genetic relation also was seen in an expression-based screen that revealed the presence of a highly transcribed CRISPR system in it,^13^ similar to the one in *Synechocystis* 6803.^14^ Moreover, the strain 6714 also represents an established laboratory strain, amenable to genetic manipulation.^15,16^

Here, we focus on the draft genome analysis of *Synechocystis* 6714 in comparison to strain 6803. In a parallel study, we will provide the primary transcriptomes of both strains under 10 different conditions using strand-specific cDNA sequencing.

## 2. Materials and methods

### 2.1. Genome sequencing, assembly, gap closure, and annotation

*Synechocystis* 6714 was purchased from the Pasteur Culture Collection (PCC) in Paris, France. Genomic DNA was extracted as described earlier.^9^ We prepared two libraries for sequencing, one with fragment lengths of 160 nt for paired-end sequencing and one with ∼3 kb long fragments, which was used for preparing a mate pair library (Illumina Mate Pair Library Prep Kit, catalogue no. PE-112-2002). Both libraries were subjected to paired-end sequencing, yielding 135 969 158 reads of 101 nt length. The accumulated sequence information resulted in a nearly 2000-fold coverage when expecting a genome of 3.5 Mb. The reads were assembled with velvet^17^ using a kmer-length of 85, a coverage cut-off at 5, and an expected coverage of 1300. This resulted in 74 contigs arranged in five scaffolds longer than 10 000 nt. Gaps within scaffolds were analysed by polymerase chain reaction (PCR) and subsequent Sanger sequencing and, if successful, closed with the obtained sequence information. This removed 19 gaps reducing the number to 55. Gene prediction and annotation was done with RAST^18^ and led to the prediction of 3733 protein coding genes, 40 tRNAs, and 1 cluster of ribosomal RNAs.

### 2.2. Orthologue prediction

Orthologue prediction was based on the 3733 open reading frames (ORFs) from *Synechocystis* 6714 and 3683 ORFs from *Synechocystis* 6803, resulting from a combination of all ORFs from Mitschke *et al.*, Supplementary data file S1^19^ and all plasmid-located ORFs annotated in CyanoBase^5^ [accession nos. AP004311, AP004312, AP004310, AP006585, L13739, L25424, and pCC5.2 (without accession no.)]. Orthologues of protein coding genes were identified using a reciprocal best blast hit (RBH) strategy. For the identification of gene families and unique genes, we used Markov clustering (MCL)^20^ on the results of reciprocal BlastP searches. Clustering into protein families using the MCL algorithm yielded 2413 shared protein families with 3385 and 3187 members in *Synechocystis* 6803 and 6714, respectively. Putative transposase genes were identified by BLASTp searches against the ISfinder^21^ requiring a BLASTp value of ≤1e10^−8^.

### 2.3. Physiological experiments

In addition to *Synechocystis* 6714, we used *Synechocystis* 6803 substrain ‘PCC-M’^9^ for comparative physiological experiments. Liquid cultures were grown at 30°C in liquid BG11 medium^12^ under continuous white light illumination of 50–80 µmol quanta m^−2^ s^−1^. For growth on solid medium, BG11 was supplemented with 0.9% agar (Kobe I, Roth, Germany). Salt-dependent growth, GG contents, and mRNA patterns were measured for cultures of 300 ml volume that were grown under constant shaking in 500 ml Erlenmeyer flasks. The cells were pre-cultivated for 2 days at standard conditions before sterile, crystalline NaCl was added to a final concentration of 2, 4, and 6% (w/v), respectively. After 4 days, 50 ml of cells was harvested by rapid filtration on hydrophilic polyethersulfone filters (Pall Supor 800 Filter, 0.8 µm). The adherent cell material was immediately dissolved in 1 ml of PGTX solution^22^ and total RNA was extracted as described.^13^ For the measurement of glucosylglycerol content, 2 ml of cells was harvested by centrifugation and soluble metabolites were extracted with 80% ethanol (HPLC gradient, Roth, Germany). The supernatant was freeze-dried. The cell extract was then purified from insoluble material by centrifugation and the supernatant was also freeze-dried. Both, the dried cell extracts and external fractions were resuspended in *A. dest* (HPLC gradient, Roth, Germany), centrifuged and the supernatant was freeze-dried again. Samples were then analysed by gas chromatography as previously described.^23^

### 2.4. Northern blot analysis

For expression analysis, 3 µg of total RNA was separated on 1.5% agarose gels, transferred to Hybond-N nylon membranes by capillary blotting and cross-linked by UV-illumination. The membranes were hybridized with ^32^P-labelled RNA probes generated from specific DNA templates by using Ambion^®^ MAXIscript^®^ T7 *In Vitro* Transcription Kit as described earlier.^24^ The oligonucleotide sequences used for the generation of DNA templates by PCR are given in Supplementary Table S1. Signals were visualized with the Personal Molecular Imager FX system and Quantity One software (Bio-Rad).

## 3. Results and discussion

### 3.1. Draft genome of Synechocystis sp. PCC 6714

The genome of *Synechocystis* 6714 was sequenced using two libraries of different lengths by paired-end sequencing and assembled into five scaffolds ranging from 46 504 to 2 984 476 nt in length. The DNA is characterized by an average GC content of 47.37%, which is very close to the value of 47.4% reported for strain 6714 in 1971 based on CsCl density gradient equilibrium centrifugation.^11^ The longest scaffold C2 likely represents the major part of the chromosome, since it closely resembles the chromosome of strain 6803. Table 1 summarizes the main features of the draft genome. Since the scaffolds C0 and C4 carry tRNA genes and the majority of their protein-coding genes have orthologues on the chromosome of *Synechocystis* 6803, they also are likely part of the chromosome. This assumption also is in line with their 3 and 5% higher GC content compared with the scaffolds C1 and C3. For comparison, the GC content of the *Synechocystis* 6803 chromosome is 47.72%, whereas it also is lower for three out of the four large plasmids (pSYSX, 42.72%; pSYSA, 44.48%, pSYSM, 42.95%).^25^ By combining the scaffolds C0, C2, and C4, we estimated the size of the *Synechocystis* 6714 chromosome to be around 3.45 Mb, which is fairly similar to the 3.57 Mb of *Synechocystis* 6803.

**Table 1.** Summary of the *Synechocystis* 6714 draft genome main features

Scaffold	Size (nt)	Genes	GC %	tRNA genes
C0	189 995	183	47.79	2
C1	181 306	201	42.41	0
C2	2 985 628	3034	47.72	35
C3	46 504	45	44.25	0
C4	287 195	270	47.13	4
Total	3 690 628	3733	47.37	41

**Figure 1.**
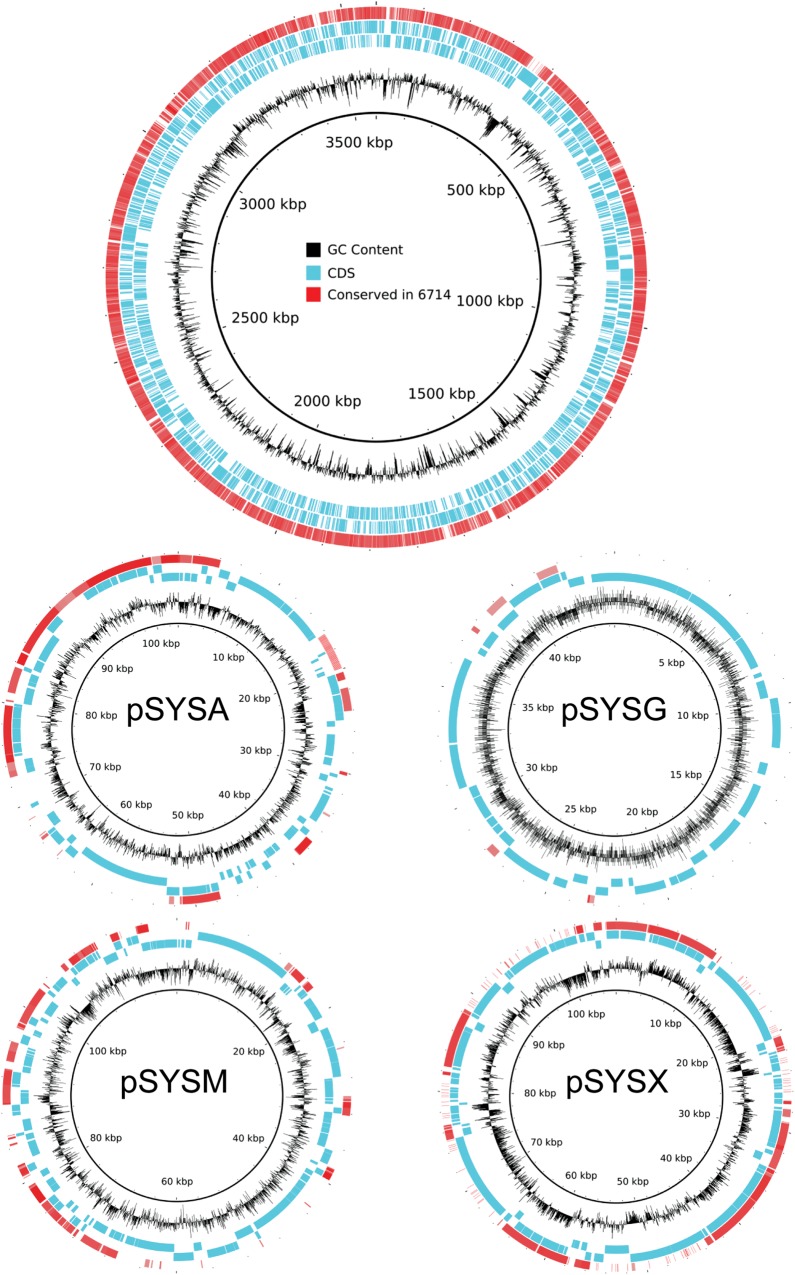
Genome coverage based on circular genome plots of the *Synechocystis* 6803 chromosome and its four large plasmids pSYSA, pSYSG, pSYSM ,and pSYSX. Tracks from the outside show (1) regions with BLASTN hit in *Synechocystis* 6714 and identity between 50% (grey) and 100% (red); (2) CDS features from forward and reverse strand in *Synechocystis* 6803; (3) GC content.

Compared with the rather high similarity of the chromosome size and coding capacity, the similarities of plasmid sequences were rather low between the two strains. Among the seven plasmids^25–28^ of *Synechocystis* 6803, we found no significant similarities toward its plasmids pSYSG, pCC5.2, pCA2.4, and pCB2.4 in strain 6714. In contrast, sequences resembling about one-third each of pSYSA, pSYSM, and pSYSX of *Synechocystis* 6803 were detected in strain 6714 (Fig. 1). Thus, our draft genome points at a different composition or lower coding capacity of extrachromosomal plasmids in strain 6714 compared with strain 6803.

A marked difference between both strains exists in the number and types of mobile genetic elements (Table 2). *Synechocystis* 6803 possesses at least 134 genes encoding transposases. These transposases, which were identified by BLASTp searches against the ISfinder database,^21^ requiring a BLASTp *E*-value of ≤10^–8^, were assigned to 11 different families, each containing 1–45 identical copies. The highest copy numbers were found for the IS630, IS5, and IS701 families of IS elements (Table 2, Supplementary Table S2). In *Synechocystis* 6714, we identified only 32 transposase genes, which belong to only six different families. The highest copy numbers were found for the IS200/IS605 family and as before in strain 6803 for IS630 and IS5 families (Table 2, Supplementary Table S3). At a first glance, this high divergence in the numbers and types of insertion sequences appears surprising, given the otherwise close relatedness among the two strains. However, this finding is in line with reports for the ISY203 group of elements (belonging to the IS4 family) that vary even among substrains of 6803. Four members of this IS element with identical nucleotide sequences were present only in the ‘Kazusa’ substrain, whereas they were absent in the genomes of other substrains.^29^

**Table 2.** Types and numbers of IS elements found on basis of identified transposase genes

IS element	Strain 6803	Strain 6714
IS1	10	0
ISTcSa	3	0
IS3	1	0
ISL3	3	0
IS4	14	0
IS200/IS605	1	5
IS256	2	1
IS5	31	7
IS630	45	11
IS701	23	3
Tn3	0	2
ISLre2	1	0
Total	134	32

Using RBH, we identified 2838 orthologous protein-coding genes between *Synechocystis* 6714 and 6803, leaving 845 specific genes in strain 6803 and 895 specific genes in strain 6714. Thus, among the two strains, more than 75% of the genes are conserved. However, many of the strain-specific genes belong to gene families that were clustered as paralogues to pairs of orthologue genes when using the MCL algorithm,^20^ indicating gene duplications, sequence, and probably also functional diversification. The full list of orthologue and paralogue genes between the two strains is presented in Supplementary Table S4. In Supplementary Tables S5 and S6, we present the lists of unique protein-coding genes. Only 537 of the 845 *Synechocystis*-6803-specific genes are located on the chromosome (=16% of all chromosomal protein-coding genes; Supplementary Table S5), whereas 308 of the genes lacking a clear orthologue (=76% of all plasmid-located protein-coding genes in 6803) can be explained by the strong differences in the plasmid-located gene pool. Moreover, it should be noted that the majority of strain-specific genes encodes for proteins of unknown function, i.e. the functional significance of the majority of differences is thus uncertain.

### 3.2. Large-scale differences between Synechocystis 6803 and Synechocystis 6714: unique genetic arrangements in a large genomic island and prophage Psy1

**Figure 2.**
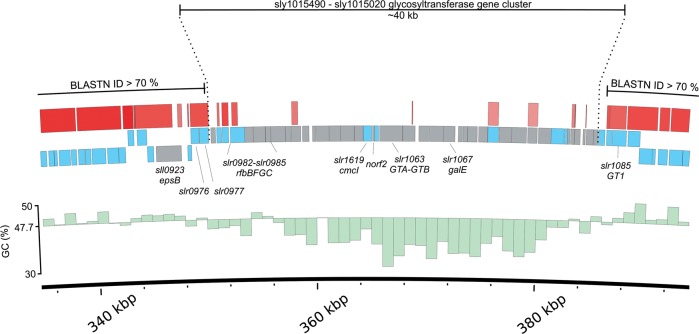
A likely genomic island in two *Synechocystis* strains. A genomic segment of ∼40 kb from *Synechocystis* 6803 is shown with some genes annotated for orientation (EPS, exopolysaccharide export protein; CmcI, Cephalosporin hydroxylase protein; GT1, GT1 family of glycosyltransferases; GTA-GTB, fusion protein joining a glycosyltransferase family A with a glycosyltransferase family B domain; Norf2 is a 68 amino acid peptide-encoding gene originally predicted on basis of transcriptome data indicating the presence of an mRNA for this conserved reading frame).^19^ Adjacent genes to this region are in the two strains of the gene pairs *slr0976/slr0977* and *sly1015510/sly1015500* encoding a DUF820 protein and an ABC transporter permease component; left side in 6803) and *slr1084/slr1085* and *sly1015040/sly1015030* (encoding a WcaF-type acyl transferase and a glycosyltransferase; right side in 6803). The GC % content, indicated by the green bars (each representing 1000 nt), drops considerably within this region. Thus, this region has features of a genomic island. The nucleotide identity to matching segments in the *Synechocystis* 6714 genome is colour coded (red >90%, light red >70%). The corresponding stretch in the *Synechocystis* 6714 genome encompasses genes *sly1015490– sly1015020*, almost entirely belonging to the list of unique genes in that strain (Supplementary Table S6). The proteins encoded by these genes are annotated as hypothetical proteins, UDP-glucose 4-epimerase, several different glycosyltransferases, rhamnogalacturonides degradation protein RhiN, dTDP-glucose 4′6′-dehydratase, methylase/methyltransferase, ABC transporter, GDP-mannose 4′6′dehydratase and as NAD-dependent epimerase/dehydratase.

The higher number of transposon genes in *Synechocystis* 6803 is correlated with a low degree of syntheny between the two strains. Another situation exists with the *rfb-*gene cluster that differs entirely between the two strains and encodes several glycosyltransferases possibly involved in cell wall biosynthesis and the modification of cell surface properties. This region has features of a genomic island, since the adjacent genes are conserved between the two *Synechocystis* strains, but the GC content drops considerably (from 48 to 35%) within this region in both strains (Fig. 2). Genomic islands consist of sets of genes that become laterally transferred, belong to the flexible gene pool of a bacterial phylum and frequently provide a certain fitness advantage.^30^ Accordingly, the most closely related homologues matching to these proteins are found in a wide variety of organisms. For the 50 genes located in the *Synechocystis* 6714 *rfb* gene cluster, the phylogenetically top-matching proteins belong to groups as diverse as Zetaproteobacteria, Bacilli, Clostridia, Armatimonadetes, Rhodopirellula, and Stigonematales cyanobacteria. The top-matching proteins against the *Synechocystis* 6803 *rfb* gene cluster proteins are of comparable diversity. A particular example is also the *norf2* gene which was annotated on the basis of transcriptome data.^19^ The most closely related proteins to Norf2 (Fig. 2) are annotated in *Thiocapsa marina* (69% identical and 86% similar residues) and several *Thioalkalivibrio* species, pointing further to the alien origin of this genomic region.

An example for genome scrambling worth mentioning exists in the hydrogenase operon that encompasses the seven genes *sll1220–sll1226* (*hoxEFUYH* plus two additional genes for proteins of unknown function) in strain 6803. In *Synechocystis* 6714, the orthologues of these seven genes (*sly1009900–sly1009960*) form a cluster with gene *sly1009870* encoding the NiFe hydrogenase metallocenter assembly protein HypD, whereas the homologue in *Synechocystis* 6803, *slr1498*, is located 1.62 Mb away.

**Figure 3.**
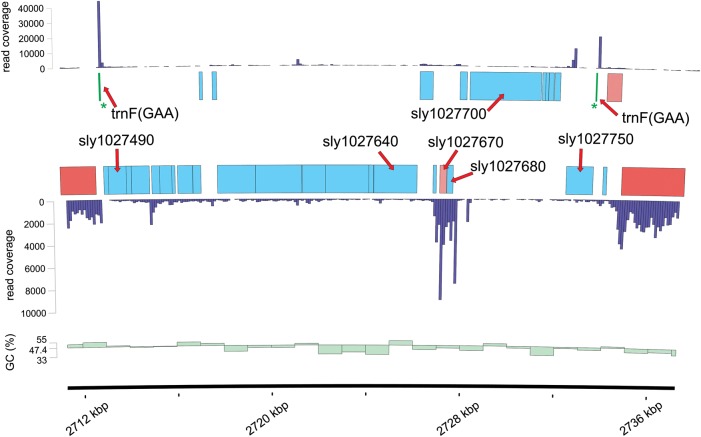
Prophage Psy1 inserted into the trnF^GAA^ gene (labelled by the green stars) of *Synechocystis* 6714. Genome position is drawn along the *x*-axis. Protein coding genes are shown in red if conserved in *Synechocystis* 6803 and in blue if not; trnF^GAA^ is shown in green. Transcriptome read counts per 100 million for the forward strand are plotted above and for the reverse strand below the CDS features. The GC % content is indicated by the green bars (each representing 1000 nt). The following genes were annotated as coding for bacteriophage-related proteins and are mentioned in the text: *sly1027640*, HK97 family phage portal protein; *sly1027490*, bacteriophage lysozyme-like protein; *sly1027670* and *sly1027680,* remotely similar to bacteriophage Cro repressor; *sly1027700*, D5 N terminal like domain-containing protein of phage D5 proteins and bacteriophage P4 DNA primases; *sly1027750*, phage integrase.

A further difference between *Synechocystis* 6714 and 6803 genomes is the presence of a prophage in the former but its lack in the latter (Fig. 3). As this prophage has not been previously described, we called it Psy1, for prophage in *Synechocystis* 1. The genomic DNA of Psy1 has integrated into the *trnF* (phenylalanine-specific tRNA^GAA^) gene, duplicating its 3′ half but restoring the gene to be functional intact. This insertion might have occurred only recently as the duplicated segment of the *trnF* gene is still sequence identical with the original prophage host gene. Although the Psy1 genome is with a total length of 20 660 nt quite short for a prophage, genomes of comparable size have recently been reported for siphoviruses, which infect marine cyanobacteria (e.g. S-CBS1 infecting *Synechococcus* strains CB0201, CB0204, CB0202, and CB0101).^31^ The annotation of Psy1 adds another 27 genes unique for strain 6714 (Supplementary Table S6). Most of these genes have no closely related homologues in database searches, indicating that Psy1 might belong to a novel group of bacteriophages. Clear homologues exist for Sly1027750, an integrase with several homologues in other cyanobacterial genomes; Sly1027640, an HK97 family phage portal protein with the tail sheath protein from the *Pseudomonas* transducing phage PhiPA3 as the best matching protein in the bacteriophage database (BlastP *E*-value 7e^−16^);^32^ Sly1027490, a lysozyme superfamily protein with the putative endolysin from *Acinetobacter* phage phiAC-1 as the best matching bacteriophage protein (BlastP *E*-value 2e^-30^);^33^ Sly1027700, a D5 N terminal like domain-containing protein of phage D5 proteins and bacteriophage P4 DNA primases (Fig. 3).

Our complementary transcriptome data (unpublished) indicate that the Psy1 genes are not significantly expressed except for a short region, encompassing the two short genes *sly1027670* and *sly1027680* (Fig. 3). One of the two proteins encoded by these two genes, Sly1027680, has similarity to bacteriophage repressor proteins and belongs to the HTH XRE family of Cro/CI repressor proteins, suggesting its possible involvement in silencing Psy1 activity. The other protein, Sly1027670, possesses a predicted partial endoribonuclease Y domain and revealed in database searches several good matches, with protein Ssl7074 from *Synechocystis* 6803 as the top hit (47% identical and 59% similar positions). Interestingly, gene *ssl7074* in *Synechocystis* 6803 is located within the CRISPR2-associated region of *cas* genes next to the *cas 6-2b* gene, a candidate for an endonuclease involved in CRISPR crRNA maturation.^14^

### 3.3. Genetic differences with particular physiological relevance

Several of the strain-specific genes likely affect the physiology or provide certain strain-specific characteristics allowing their settlement in specific environmental niches (see Supplementary Table S5 and S6 for the list of unique genes in *Synechocystis* 6803 and 6714, respectively). For instance, only *Synechocystis* 6803 possesses genes for the proteins Flv2 and Flv4, which are essential for growth under fluctuating light and are supposed to protect photosystem II against photoinhibition.^34^ In contrast, in *Synechocystis* 6714, two operons are found, each encoding all subunits of the high-affinity K^+^ transporter Kdp,^35^ similar to the situation in filamentous cyanobacteria such as *Anabaena* sp. PCC7120, whereas *Synechocystis* 6803 harbours only one copy of the *kdp2* type.^36^ To date, it was believed that unicellular cyanobacteria have a single *kdp* system or none, whereas filamentous cyanobacteria have two or more copies.^36^

A distinct group of protein-coding genes that differs between the two strains are associated with the CRISPR system, the prokaryotic immune system, accounting for 17 different proteins alone (Supplementary Tables S5 and S6). There are three distinct loci of CRISPR-*cas* genes in both strains.^13,14^ One of them (called CRISPR3/CRISPR3*) is highly conserved, whereas the other two appear to have been substituted over their entire length, possibly by an active mechanism of exchange. Details of the different CRISPR-*cas* loci were published separately.^13^

**Figure 4.**
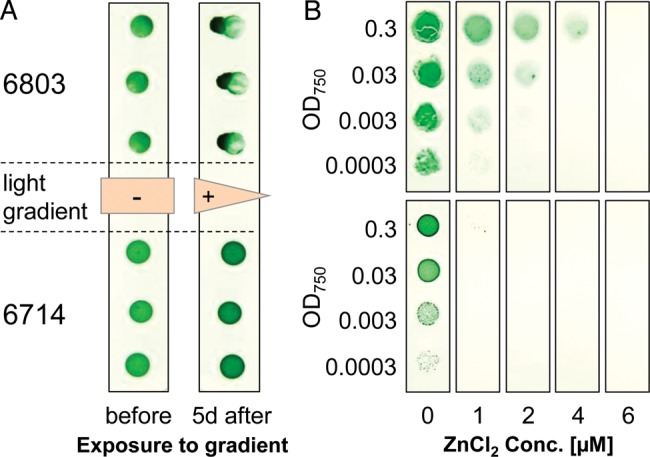
Verification of physiological and genetic differences predicted upon draft genome analysis of *Synechocystis* 6714. (A) Phototactic motility of *Synechocystis*. Cells from liquid cultures (OD_750_ = 0.2) were dropped onto a BG11-agar plate, pre-cultivated under standard conditions for 3 days and afterwards exposed to a gradient of incident light with intensity 50 µE. The photograph was taken before and after further 5 days. (B) Drop dilution assay showing the growth on solid media in the presence of increasing concentrations of Zn^2+^ ions. The photograph was taken after 10 days of standard cultivation.

One feature that has been reported to differ even between *Synechocystis* 6803 substrains is motility. Therefore, a standard motility assay was conducted and demonstrated that *Synechocystis* 6714 is non-motile (Fig. 4). However, among the known mutations that affect motility in *Synechocystis* 6803 substrains, we found an intact *spkA* protein kinase gene,^37^ an intact *hfq* gene,^38^ as well as most *pil* genes.^39^ However, one missing gene in *Synechocystis* 6714 encodes an orthologue of PilA5 (*slr1928* in *Synechocystis* 6803), a type 4 pilin-like protein, which is involved in the formation of thick pili and motility^40^ and therefore may explain the observed phenotype.

Furthermore, a gene cassette involved in the sensing and the resistance to Zn^2+^ and Co^2+^ (including the genes *corR, corT, ziaA,* and *ziaR*; Supplementary Table S5)^41,42^ appears to be specific for *Synechocystis* 6803 and missing in strain 6714. The functional significance of this difference was tested in growth experiments in the presence of increasing amounts of Zn^2+^ ions and revealed the higher tolerance of strain 6803 against high Zn^2+^ levels (Fig. 4).

**Figure 5.**
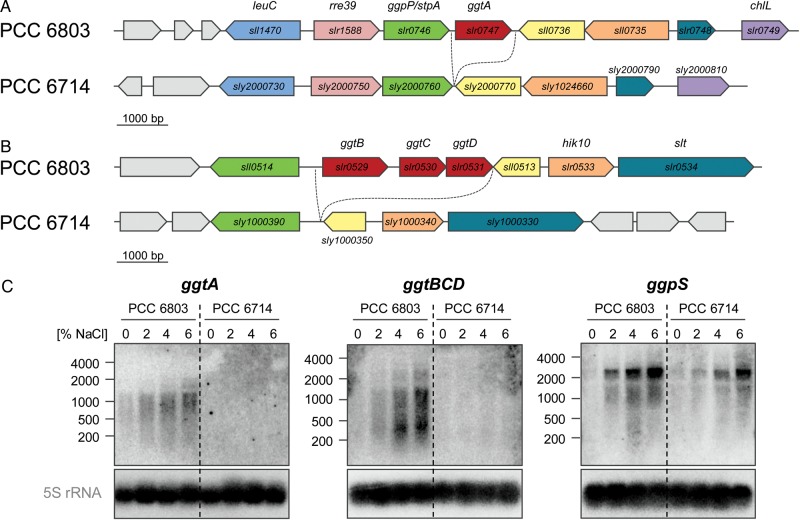
Comparative genome analysis reveals the absence of the genes encoding the glucosylglycerol transport system (Ggt). (A) Genomic region encompassing the *ggtA* gene in *Synechocystis* 6803 and of the corresponding region in *Synechocystis* 6714. (B) Genomic region encompassing the *ggtBCD* operon. Apart from Ggt, both loci are well conserved (protein identity scores of ca. 90%). (C) Occurrence of mRNAs for *ggtA*, *ggtBCD*, and *ggpS* in salt-treated cells of both strains. For *ggpS*, salt-dependent expression was observed in both strains, whereas mRNAs for *ggtA* and *ggtBCD* were not detected in strain 6714.

Another four genes, which were not found in the *Synechocystis* 6714 genome, are the *ggtABCD* genes encoding a transport system for the (re-)uptake of the compatible solute glucosylglycerol.^43,44^ Apart from that, the loci adjacent to *ggtA* or *ggtBCD* in *Synechocystis* 6803 are conserved in the genome of *Synechocystis* 6714 (Fig. 5A and B). To verify the absence of Ggt, Northern hybridization with ^32^P-labelled probes specific for *ggtA* or *ggtBCD* was performed with RNA from salt-treated cells. As expected, no mRNA was detected in salt-treated cells of strain 6714, whereas the expression level of the *ggt* genes correlated with the external salinity in *Synechocystis* 6803 (Fig. 5C). Moreover, the relative abundance of the mRNA for *ggpS*, the gene encoding the key enzyme of glucosylglycycerol synthesis, the main compatible solute in these two strains, was measured and revealed its salt-dependent expression in *Synechocystis* 6714 (Fig. 5C), similar to the well-characterized situation in *Synechocystis* 6803.^45^ These results further substantiated that, even though the 6714 genome is not completely finished, the lack of certain genes correlates to physiological differences.

**Figure 6.**
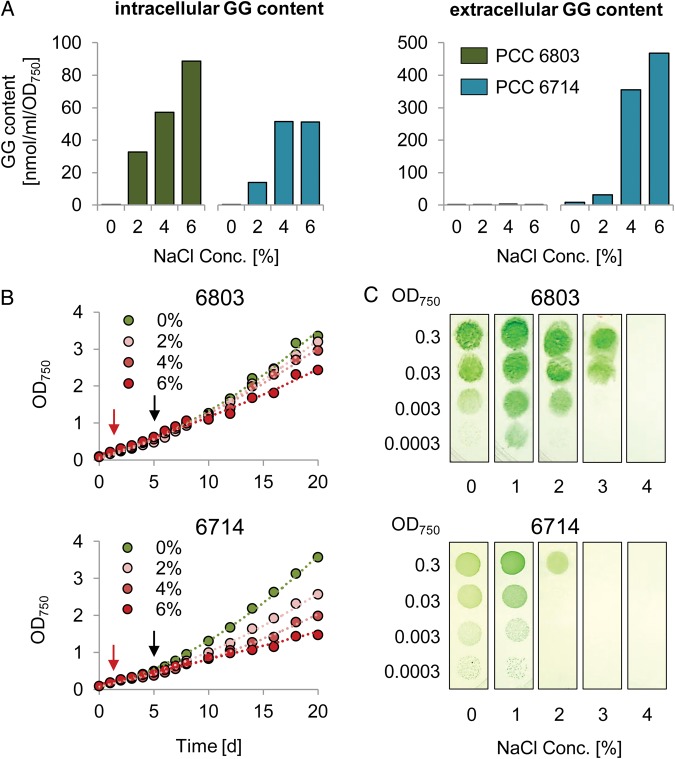
Effects of the absence or presence of the *ggtABCD* system. (A) Measurement of intracellular and extracellular GG content in the two *Synechocystis* strains. (B) Long-term growth of *Synechocystis* 6803 and 6714 in liquid cultures under salt stress. Cells were pre-cultivated without salt for 2 days before salt was added to a final concentration of 2, 4, and 6% (w/v), respectively (time point is marked by a red arrow). After further 4 days, samples for RNA extraction and GG measurements were taken (marked by a black arrow). The data are representatives of two independent experiments. (C) Drop dilution assay illustrating the growth on solid media in the presence of increasing NaCl concentrations. Cell material of exponentially growing cultures was diluted to an OD_750_ of 0.3 and 20 µl of this suspension as well as a dilution series were dropped on NaCl-containing, agar-solidified BG11 medium. The photograph was taken after 10 days under constant illumination of 50–60 µE.

Deletion of Ggt in *Synechocystis* 6803 results in the inability of taking up GG as well as trehalose and sucrose.^43,44^ Furthermore, the *ggtA* mutant of strain 6803 became leaky for GG, i.e. an increase in GG in the medium was observed when cells were grown in salt medium, suggesting that its transport is mainly necessary for recovery of GG leaked through the cytoplasmic membrane into the periplasm.^43^ Due to the absence of Ggt in strain 6714, an uptake of GG seemed unlikely and a GG accumulation in the medium during growth at elevated salinities should be measureable. To test this hypothesis, the intra- as well as extracellular GG contents were measured for cultures acclimated to different salinities. Under freshwater conditions (0% NaCl), the cells of both strains were virtually free of GG. In *Synechocystis* 6803, the intracellular GG level increased corresponding to the external salt level, whereas in the surrounding medium, virtually no GG was found (Fig. 6A). In principle, a correlation of the internal GG content and the external salt concentration was also observed for strain 6714. Up to a salinity of 4% NaCl, the GG concentrations with respect to the average biomass (expressed as OD_750_) were similar. However, no further increase was observed when cells were grown at 6% NaCl pointing to a somewhat lower salt tolerance of strain 6714 (see below). Interestingly, GG also accumulated in high amounts in the surrounding medium, which supports the assumption that an effective system for the re-uptake of GG is missing in *Synechocystis* 6714 (Fig. 6A). Similar to the internal, also the external GG content increased according to the salinity (Fig. 6A).

The synthesis of GG is costly regarding the consumption of energy and carbon. Thus, an effective uptake system seems reasonable for a bacterium whose osmotic adaptation is based on GG accumulation. For a Ggt mutant of *Synechocystis* 6803, it was postulated that the inability to take up leaked GG should result in a lower salt tolerance or at least to a lower growth performance under higher salinities, especially if the cells are grown under Ci-limitation.^43^ Interestingly, in liquid cultures that had a rather low surface:volume ratio, which results in a poor aeration in turn leading to a low degree of Ci availability, strain 6714 grew slower compared with strain 6803 in the presence of increased NaCl concentrations (Fig. 6B). In contrast, both strains showed similar growth performance under freshwater conditions (0% NaCl). Moreover, strain 6714 also showed a lower salt tolerance when cells were grown on solid medium in the presence of various NaCl concentrations (Fig. 6C). In the presence of 3% NaCl, no colonies were observed for strain 6714, whereas 6803 grew well under the same condition.

## 4. Discussion

The here presented draft genome sequence of *Synechocystis* 6714 allows comparative genome-based studies, as we demonstrate for several examples of physiological importance. Other comparative analyses include the direct comparison of promoter elements and of conserved sRNAs with similar regulation, implying conservation of function as we are showing in a separate manuscript.

We have noticed several important differences between the two strains. As the absence of a gene from a draft genome sequence might be considered ambiguous, we have highlighted cases for which the physiological difference predicted by the lack of certain genes could indeed be demonstrated. Among these differences is the lack of a transport system for the re-uptake of the compatible solute glucosylglycerol, linked to the observation that strain 6714 showed growth retardation at salinities above 2%, whereas strain 6803 even managed 4% in liquid cultures. The accumulation of GG in the external medium meaning a permanent loss of fixed carbon might be reasonable for the reduced salt tolerance of strain 6714 as has been postulated earlier.^43^

In addition to compatible solute accumulation, a balancing of the ionic composition is also important to cope with changing salinities. For instance, an active extrusion of Na^+^ is essential for cyanobacteria in order to maintain a low, non-toxic intracellular level. Homologues for most genes known to be involved in Na^+^ transport and which might be also important during salt acclimation (for review, see Hagemann)^46^ are found in the genome of *Synechocystis* 6714. However, a homologue of *sll1685* (PxcA) which might be involved in the energetization of Na^+^ transport is missing. Furthermore, the genome of *Synechocystis* 6714 harbours two copies of the *kdp* operon each encoding a high-affinity K^+^ transporter (genes *sly5000010–sly5000050* and *sly1021590– sly1021630*), whereas strain 6803 has a single copy of this operon *(slr1728–slr1731*). The Kdp ATPase system, initially characterized in *Escherichia coli*, is responsible for the immediate uptake of K^+^ after salt or osmotic shock in *E. coli*.^35^ In combination with glutamate as an organic counter ion, K^+^ is believed to act as a temporary compatible solute and moreover as a regulatory signal for the initiation of subsequent acclimation processes, also in cyanobacteria.^46,47^ Interestingly, the kinetics for the uptake of K^+^ in cyanobacteria after salt shock was characterized for *Synechocystis* 6714.^48^ A sudden osmotic shift by adding 500 mM NaCl was followed by a transient accumulation of K^+^ which started within the first minutes, peaked at around 30–60 min and declined after 24 h to levels similar to non-shocked cells. The decrease in K^+^ was accompanied by an accumulation of GG. The kinetics of a K^+^ uptake have not been measured so far for *Synechocystis* 6803, but it might be a bit different from the process in *Synechocystis* 6714 due to the absence of a second *kdp* operon.

Another interesting observation is the putative substitution of a gene cassette of ∼40 kb encoding several glycosyltransferases, transport proteins, and hypothetical proteins in the two strains. Together with the presence of some genes not found in any other cyanobacteria and the strongly reduced average GC % content in this region, this region is likely representing a genomic island. Physiologically and ecologically important genomic islands have been identified in several marine cyanobacteria.^2,3,49,50^ Interestingly, glycosyltransferase and glycoside hydrolase gene families have also been found frequent in several of these cyanobacterial genomic islands. Therefore, the modification of cell surface polysaccharide and lipopolysaccharide biosynthesis by several of these enzymes, presumably allowing diversification of cell surface features appears central for this group of organisms. Such modification capacity is likely to be relevant in the avoidance of grazers and even more in the avoidance of bacteriophage infection.^51^

In conclusion, the draft genome analysis of *Synechocystis* 6714 allows to follow interesting research problems in this strain. However, most importantly, it opens exciting new opportunities when working with the most advanced cyanobacterial model, *Synechocystis* 6803.

## 5. Data access

The assembled scaffolds of the *Synechocystis* sp. PCC6714 genome are available under the accession no. AMZV01000000 at Genbank. The annotated version including also short assembled regions is available at http://www.cyanolab.de/Supplementary.html.

## Supplementary Data

Supplementary Data are available at www.dnaresearch.oxfordjournals.org.

## Funding

The research leading to these results has received funding from the Federal Ministry of Education and Research grant ‘e:bio RNAsys’ 0316165 (to W.R.H.).

## Supplementary Material

Supplementary Data
