# Supplementary material for: Comparative Genome Analysis of the Closely Related Synechocystis Strains PCC 6714 and PCC 6803
Source: DNA Res. 2014 Jan 9;21(3):255–66. doi: 10.1093/dnares/dst055 (PMC4060947; doi:10.1093/dnares/dst055)
Supplement: Supplementary Data [file supp_21_3_255__index.html]

Comparative Genome Analysis of the Closely Related Synechocystis Strains PCC 6714 and PCC 6803 — Supplementary Data 

# Comparative Genome Analysis of the Closely Related *Synechocystis* Strains PCC 6714 and PCC 6803

## Supplementary Data

Supplementary Data

**Files in this Data Supplement:**

- Supplementary Tables - xlsx file
